# Dielectrophoresis for Isolating Low-Abundance Bacteria Obscured by Impurities in Environmental Samples

**DOI:** 10.1007/s10126-025-10441-0

**Published:** 2025-03-14

**Authors:** Jaeyoung Yu, Hajime Yuasa, Ikuo Hirono, Keiichiro Koiwai, Tetsushi Mori

**Affiliations:** 1 Department of Biotechnology and Life Science, Tokyo University of Agriculture and Technology, 2-24-16 Naka-Cho, Koganei-Shi, Tokyo, 184-8588 Japan; 2 Laboratory of Genome Science, Tokyo University of Marine Science and Technology, Konan 4-5-7, Minato-Ku, Tokyo, 108-8477 Japan

**Keywords:** Dielectrophoresis, Host-associated bacteria, Host-microbial interactions, Impurity reduction, Low-abundance bacteria

## Abstract

Bacterial communities associated with living organisms play critical roles in maintaining health and ecological balance. While dominant bacteria have been widely studied, understanding the role of low-abundance bacteria has become increasingly important due to their unique roles, such as regulating bacterial community dynamics and supporting host-specific functions. However, detecting these bacteria remains challenging, as impurities in environmental samples mask signals and compromise the accuracy of analyses. This study explored the use of dielectrophoresis (DEP) as a practical approach to isolate low-abundance bacteria obscured by impurities, comparing its utility to conventional centrifugation methods. Using two shrimp species, *Neocaridina denticulata* and *Penaeus japonicus*, DEP effectively isolated bacterial fractions while reducing impurities, enabling the detection of bacteria undetected in centrifuged samples. These newly detected bacteria were potentially linked to diverse ecological and host-specific functions, such as nutrient cycling and immune modulation, highlighting DEP as a highly potential approach to support the study of host-microbial interactions. Overall, we believe that DEP offers a practical solution for detecting overlooked bacteria in conventional methods and exploring their diversity and functional roles, with potential contributions to aquaculture and environmental biotechnology.

**Supplementary Information:**

The online version contains supplementary material available at 10.1007/s10126-025-10441-0.

## Introduction

Living organisms host numerous bacteria, some of which provide beneficial advantages, while others may exhibit pathogenic properties. Beneficial bacteria in aquatic organisms contribute to health and ecosystem balance by aiding digestion and nutrient acquisition (Holt et al. 2021) while producing bioactive compounds to combat pathogens (Diwan et al. 2023). In contrast, pathogenic bacteria can infect the host, potentially causing acute diseases and, in severe cases, contributing to mortality (Mkulo et al. 2024). Therefore, elucidating the roles and functions of these host-associated bacteria can provide significant insights into both basic and applied sciences. In particular, understanding the symbiotic relationships between hosts and their microbiota enables practical applications, such as the development of probiotics to improve aquaculture productivity and sustainability (Muthu et al. 2024). Furthermore, discovering new beneficial bacteria with unique metabolic capabilities can contribute to biotechnological advancements in bioremediation and the development of novel antibiotics (Diner et al. 2024; Burbick et al. 2024). On the other hand, the identification of pathogenic bacteria is also crucial for implementing effective disease prevention and management strategies in aquaculture, where health is closely linked to environmental and bacterial factors (Okon et al. 2023).

Over the past decades, research on host-microbial interactions has primarily focused on the role and importance of dominant bacteria. However, in recent years, there has been growing interest in understanding the roles and significance of low-abundance bacteria, which can perform specific functions within the host despite their low presence (Han and Vaishnava 2023). Similar to the roles of dominant bacteria, low-abundance bacteria have also been demonstrated to provide beneficial effects, such as maintaining bacterial community balance (Benjamino et al. 2018) and supporting host-specific functions, including immune development (Alexander et al. 2022) or disease prevention (Herp et al. 2021). On the other hand, certain low-abundance bacteria have been identified as potential pathogens in diverse environments (Hamilton et al. 2018; Sun et al. 2024; Li et al. 2024). Some of these bacteria can exhibit opportunistic properties, residing in the host without causing disease but inducing infections when the host’s immune system is compromised or when they access sterile parts of the host’s body (Wu et al. 2021). From providing beneficial effects to exhibiting opportunistic pathogenicity, these characteristics of low-abundance bacteria underscore their diverse and complex roles within the host ecosystem.

As the ecological and physiological significance of low-abundance bacteria has become increasingly recognized, advanced methods such as metagenomic sequencing have been introduced, significantly improving the detection of these bacteria beyond traditional culture-based approaches (van Dijk et al. 2022). Despite these advancements, several challenges still hinder a comprehensive understanding of low-abundance bacterial communities. For instance, issues such as the uneven distribution of low-abundance bacteria, increased background noise in samples (e.g., non-target DNA from host cells or free environmental DNA), and reduced data reproducibility due to environmental variability have been frequently highlighted as major obstacles (Kang and Mills 2006; Pistone et al. 2021; Du et al. 2022). In many cases, these challenges are aggravated by the presence of impurities such as organic debris and minerals, which create complex matrices that amplify background noise and subsequently hinder the accurate detection and characterization of low-abundance bacteria (Wilpiszeski et al. 2019; Fatoyinbo et al. 2014). While this underscores the importance of adequate sample preparation, conventional methods such as centrifugation and filtration remain widely used. Although these methods are partially effective, they often leave residual impurities that obscure signals from low-abundance bacteria (Vaishampayan et al. 2010; Nnadozie et al. 2015), thus highlighting the need for alternative approaches to efficiently separate bacteria from impurities.

To address this, we explored the use of dielectrophoresis (DEP) as an approach to isolate low-abundance bacteria that were undetectable by conventional methods. DEP uses non-uniform electric fields to manipulate and capture polarized particles (Wakizaka et al. 2020), enabling the separation of bacteria from impurities in environmental samples (Yu et al. 2024). In this study, we utilized two shrimp species, the freshwater shrimp, *Neocaridina denticulata*, and the target species for marine aquaculture, Kuruma shrimp *Penaeus japonicus*, as models for aquatic organisms to compare bacterial diversity and composition in fractions obtained through centrifugation and DEP. Our findings suggest that DEP represents a promising tool for uncovering low-abundance bacteria obscured by impurities, offering new opportunities to advance our understanding of host-microbial interactions.

## Materials and Methods

### Shrimp Maintenance and Preparation of Bacterial Fraction

The Neocaridina shrimps, *Neocaridina denticulata*, were purchased from a shrimp breeder and maintained in an aquarium with fresh water circulation and filtration at 22–26 °C. The Kuruma shrimps, *Penaeus japonicus*, were purchased from a local farmer and kept in artificial seawater with a recirculating water system, maintained at 25 °C with a salinity of 30–35 ppt. Both shrimps were fed daily: the Neocaridina shrimps were provided with specified food (JAN: 4971453054048; Itosui. Co. Ltd., Tokyo, Japan), while the Kuruma shrimps were fed with specified Kuruma shrimp food (Goldprawn; Higashimaru. Co. Ltd., Kagoshima, Japan).

Due to the small size of the Neocaridina shrimps, three whole individuals were directly processed and pooled. In contrast, the larger Kuruma shrimps allowed for the dissection of specified organs, including the gills (left and right), stomach, and gut, from two individuals, with samples for each organ type pooled and analyzed accordingly. To minimize external contamination, the Neocaridina shrimps were rinsed with 1 × Phosphate-Buffered Saline (PBS; Thermo Fisher Scientific, Tokyo, Japan), while the Kuruma shrimps were wiped with alcohol before dissection. The extracted organs from the Kuruma shrimps were placed in filter-sterilized seawater and kept at 4 °C until processing. The prepared samples were transferred to a 2.0 mm ZR BashingBead Lysis Tube (Zymo Research, CA, USA) containing 500 μL of 1 × PBS. The transferred samples were disrupted using a FastPrep-24™ 5G (MP Biomedicals, Chiba, Japan) for 40 s at a speed of 6 m/s. The disrupted samples were transferred to 1.5 mL centrifuge tubes and centrifuged at 7500 × g for 10 min. The supernatant was discarded, and the resulting pellets were resuspended in 1 × PBS to an OD_660_ of 1.0, to attain the final bacterial fraction.

### DEP Capture for Obtaining Bacterial Fractions with Reduced Impurities

Bacterial fractions with reduced impurities were obtained using DEP based on a previous report (Yu et al. 2024). Briefly, 100 μL of the final bacterial fraction was reconstituted in 1 mL of ELESTA-PBS (EP) buffer (200:1 mixture of ELESTA buffer (ELB100N; AFI Corporation, Kyoto, Japan) and 1 × PBS). DEP capture was performed using PixeeMo® (AFI Corporation) equipped with a D32L210 microchip (AFI Corporation). Prior to use, the microchip was equilibrated using EP buffer at a 60 µL/min flow rate for 5 min. Subsequently, the reconstituted final bacterial fraction was loaded at an 8 µL/min flow rate, and a frequency of 300, 1000, 3000, or 7000 kHz with a 20 Vpp voltage was applied. Post-capture, the initial flowthrough (waste fluid) was collected, the frequency and voltage were turned off, and the second flowthrough (DEP-captured sample) was obtained by loading the EP buffer at a 60 µL/min flow rate for 10 min. The waste fluid was reloaded through the microchip to ensure efficient bacterial capture, and the process for obtaining the DEP-captured sample was repeated. Finally, the DEP-captured sample and waste fluid were centrifuged at 7500 × g for 5 min, and the resulting pellets were resuspended in 100 μL of 1 × PBS for subsequent assessments.

### Nanopore Sequencing for Bacterial Diversity Analysis

To compare bacterial composition and diversity between the centrifuged and DEP-captured samples from the Neocaridina and Kuruma shrimps, genomic DNAs were first extracted using the DNeasy UltraClean Microbial Kit (QIAGEN, Tokyo, Japan), followed by 16S rRNA gene sequencing using a nanopore platform. A library for the 16S rRNA gene was constructed using the 16S Barcoding Kit (SQK-16S114.24; Oxford Nanopore Technologies (ONT), Oxford, UK), and nanopore sequencing was performed on a MinION Mk1B (ONT) with an FLO-MIN114 flow cell following the manufacturer’s protocol. Sequencing was stopped when sufficient reads were obtained. The raw FAST5 files were sequentially trimmed for the quality and length (*Q*-score, > 12; length, 1200–1800 bp) and base-called in the high-accuracy mode using Dorado v7.2.13 within MinKNOW v5.8.7 (Pugh 2023). The base-called raw FASTQ files were used to generate a taxonomic abundance profile of 16S rRNA using Emu v3.4.5 (Curry et al. 2022). To minimize the potential influence of sequencing noise, a 0.5% threshold was applied, and taxonomic profiles were sorted at the genus level (Rabbachin et al. 2024; Amstler et al. 2024).

### Validation of Bacterial Viability

The viability of bacteria in the DEP-captured samples, as well as those in the centrifuged and waste fluid samples, was verified using Syto 9 and PI fluorophores from the LIVE/DEAD™ *Bac*Light™ Bacterial Viability Kit (Thermo Fisher Scientific) in combination with fluorescence microscopy. Microscopic images were captured using a BX53 upright fluorescence microscope equipped with a 100 × objective lens (Olympus, Tokyo, Japan) and a DP74 digital camera. Fluorescing cells were observed using the U-FBW wideband mirror unit, allowing for simultaneous red and green fluorescence visualization. Microscopic images were minimally edited to achieve the desired sizes using Adobe Photoshop CC 2018 v19.1.3 (Adobe System, Tokyo, Japan).

### Bacterial Cultivation and Identification

Bacterial identification was conducted to evaluate the effects of impurity reduction on the diversity of bacteria cultivated. The centrifuged and DEP-captured samples were diluted in gradients ranging from 10^3^ to 10^7^ with 1 × PBS, then spread onto Trypticase Soy Broth Agar (TSB; 30 g ${\text{L}}^{-1}$ trypticase soy broth, 20 g ${\text{L}}^{-1}$ agar), and incubated at 25 °C for 48 h. For the bacterial identification, 30 single colonies were randomly selected from each plate and subjected to colony PCR using the 16S rRNA universal primers 27F (5′ – AKWGTTTGATCMTGGCTCAG) and 1492R (5′ – GGHTACCTTGTTACGACTT). The PCR reaction was carried out using the AmpliTaq Gold 360 Master Mix (Thermo Fisher Scientific) following the manufacturer’s guidelines, with amplification performed on a C1000 Touch Thermal Cycler (Bio-Rad, Hercules, CA, USA). The thermocycling protocol included an initial denaturation at 95 °C for 10 min, followed by 25 cycles of denaturation at 95 °C for 30 s, annealing at 54 °C for 30 s, and extension at 72 °C for 90 s. A final extension step at 72 °C for 7 min was added to complete the reaction. DNA sequencing of the PCR products was outsourced to FASMAC Co., Ltd., Kanagawa, Japan, and the resulting sequences were analyzed and classified using Geneious Prime v2020.2.5 (Geneious; AKL, New Zealand).

## Results and Discussion

### DEP Optimization Improves Isolation of Viable Low-Abundance Bacteria Obscured by Impurities

This study evaluated the utility of DEP for isolating low-abundance bacteria obscured by impurities in environmental samples. The freshwater Neocaridina shrimps, due to their ease of maintenance in laboratory settings, were used as a model for initial testing and optimization of DEP. In contrast, the marine Kuruma shrimps, which are extensively cultivated worldwide and hold significant economic importance (Chen et al. 2024), were selected to assess the applicability of DEP against more complex biological samples. This model selection was intended not only to establish a foundation for evaluating the potential of DEP to function against samples attained from controlled laboratory settings but also against samples from open aquaculture environments.

As an initial step, nanopore sequencing was employed to evaluate bacterial composition and diversity at varying DEP frequencies (300, 1000, 3000, and 7000 kHz) within the Neocaridina shrimp models. Detailed information on sequencing quality and length is provided in Table S1. The results showed that DEP frequency significantly impacts bacterial isolation as it corresponds to the dielectric properties of each bacterium. These properties are closely linked to bacterial characteristics such as membrane capacitance, cytoplasmic conductivity, and external structures like pili and flagella (Betts and Brown 1998). At the lower frequencies of 300 and 1000 kHz, *Aeromonas* (Table 1), which exhibits electrically conductive pili and electrogenic capabilities (Castro et al. 2014; Prados et al. 2021), was predominantly isolated. The significant proportion of *Aeromonas* (87.9%) in the centrifuged sample further corroborates this observation (Table 1), suggesting that its high initial abundance may have also influenced its selective isolation under these conditions. In contrast, higher DEP frequencies of 3000 and 7000 kHz enabled the detection of 10 and 12 bacterial genera, respectively (Table 1). This result suggests that higher frequencies enhance the polarization of bacteria with lower conductivities or different membrane compositions. Notably, among the detected genera, *Azospira*, *Chryseobacterium*, *Hyphomicrobium*, *Pseudomonas*, and *Paucibacter* were not observed in the centrifuged sample. The presence of such bacteria highlights the ability of DEP to isolate low-abundance bacteria presumably obscured by impurities within bacterial fractions prepared using conventional methods such as centrifugation.

**Table 1:** Bacterial distribution, as revealed by nanopore sequencing, following DEP capture from the Neocaridina shrimps, highlighting the effect of DEP frequency

Name of genus	Bacterial distribution (%)				
	Centrifuged sample	DEP-captured sample			
		300 kHz	1000 kHz	3000 kHz	7000 kHz
*Acidovorax*	0.6			1.4	2.0
*Aeromonas*	87.9	100	100	85.6	76.3
*Azospira*				0.7	0.7
*Candidatus Methylopumilus*	3.1			3.0	4.5
*Chryseobacterium*				0.7	1.3
*Cloacibacterium*	1.5			4.6	7.0
*Flavobacterium*	1.0			1.2	2.2
*Hyphomicrobium*				0.5	0.7
*Methylophilus*	0.6				
*Mitsuaria*	1.8				0.8
*Paucibacter*					0.6
*Pseudomonas*				0.5	0.7
*Rickettsia*	1.2			1.8	3.2
*Roseateles*	0.6				
*Vibrio*	0.5				
*Zoogloea*	1.1				
Number of detected bacterial genera	11	1	1	10	12

**Fig. 1:**
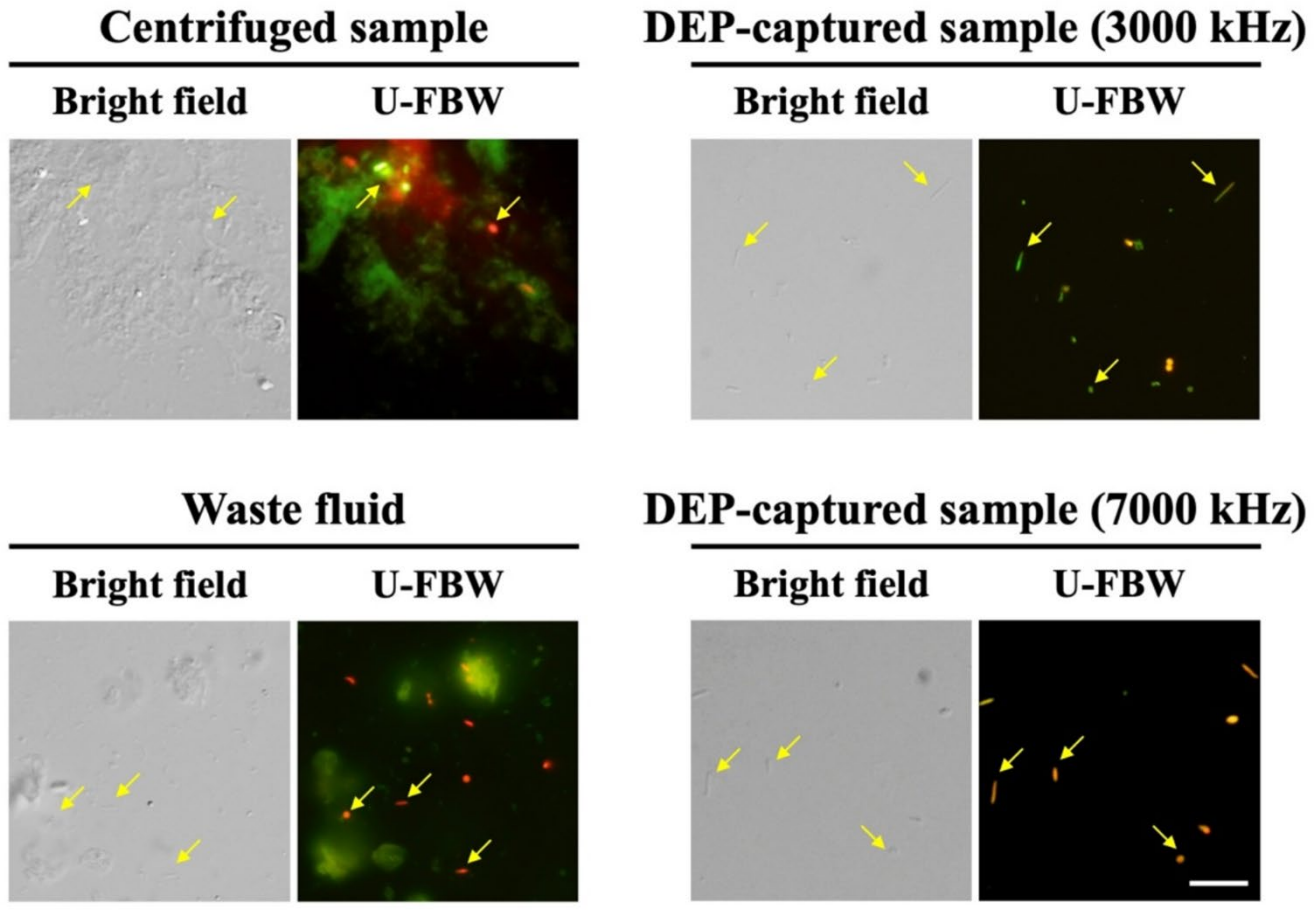
Results of the LIVE/DEAD™ BacLight™ Bacterial Viability Kit following DEP capture at 3000 and 7000 kHz from the Neocaridina shrimps. Green and red fluorescence denote viable and non-viable bacteria, respectively. The yellow arrows indicate bacteria. Scale bars are 5 μm

While higher frequencies improve the ability to isolate diverse bacteria, it is also important to ensure that the cells are viable, as it opens opportunities for downstream applications. High-frequency electric fields can destabilize bacterial membranes, causing electroporation that forms temporary or permanent pores (Napotnik et al. 2021). Unlike centrifugation, which often yields mixed fractions of viable and non-viable cells along with impurities, DEP can selectively isolate viable bacteria by leveraging their electrical properties (Yu et al. 2024; Chu et al. 2023). To reflect this aspect, we assessed the viability of bacteria isolated via DEP using Syto 9 and PI. The results showed that at 3000 kHz, green fluorescing bacteria were most prominent within the DEP-captured sample, indicating that most cells had intact membranes and preserved structural integrity (Fig. 1). In contrast, the waste fluid primarily consisted of non-viable red fluorescing bacteria and impurities exhibiting low autofluorescence. However, at 7000 kHz, orange fluorescing bacteria dominated the DEP-captured samples (Fig. 1), suggesting membrane damage and reduced viability due to excessive electrical stress (Gião et al. 2009). These results underscore the importance of optimizing DEP frequencies to balance effective bacterial isolation and preservation of bacterial integrity. Based on these findings, in this study, 3000 kHz was determined as the optimal frequency for isolating diverse and viable low-abundance bacteria with reduced impurities.

### Newly Detected Bacteria Via DEP Suggest Potential Host-Bacteria Association

Using the optimized frequency of 3000 kHz, we subsequently investigated the characteristics of newly detected bacteria via DEP. This analysis highlights the potential of these bacterial genera and their possible association with host-microbial interactions and environmental ecosystems. From the DEP-captured sample of the Neocaridina shrimps, four bacterial genera (*Azospira*, *Chryseobacterium*, *Hyphomicrobium*, and *Pseudomonas*) that were not detected in the centrifuged samples were identified (Table 1). Among these, *Chryseobacterium* and *Pseudomonas* are frequently associated with aquatic organisms as opportunistic pathogens (Chenia 2015; Korkut et al. 2018). To our knowledge, no report has directly confirmed their pathogenicity in shrimp; however, both genera have been detected in various shrimp species (Suji et al. 2014; Monghit-Camarin et al. 2020; Morita et al. 2021). Interestingly, despite their general association with pathogenicity in aquatic organisms, certain *Pseudomonas* strains are shown to inhibit pathogenic bacteria in shrimp (Chythanya et al. 2002) or promote shrimp growth via nitrate cycling (Hastuti et al. 2023), indicating strain-specific and context-dependent interactions. In contrast, *Azospira* and *Hyphomicrobium*, though less studied in aquatic organisms, have occasionally been detected in shrimp gut microbiota (Hou et al. 2024; Rungrassamee et al. 2014). Interestingly, a study demonstrated that ammonia nitrogen stress significantly reduces *Hyphomicrobium* abundance in shrimp guts (Hou et al. 2024). This suggests that *Hyphomicrobium* is sensitive to nitrogen compounds and indicates the interaction between the shrimp gut microbiota and environmental factors. Considering their role in promoting nitrogen cycling in aquatic environments (Osaka et al. 2008; Li et al. 2023b), *Azospira* and *Hyphomicrobium* may be expected to play complementary roles in mitigating environmental stressors and supporting shrimp health.

**Fig. 2:**
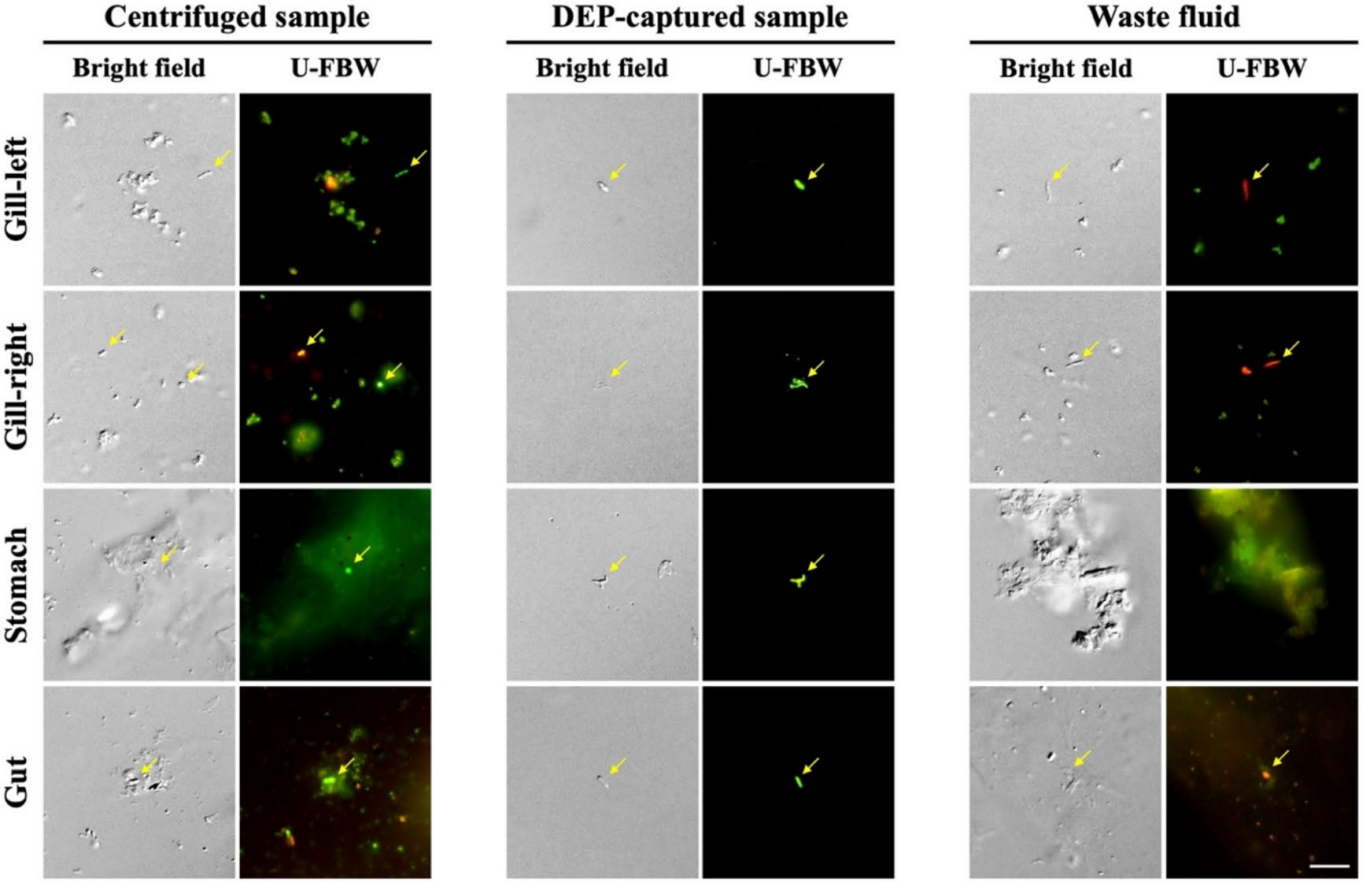
Assessment of bacterial capture efficiency using the LIVE/DEAD™ BacLight™ Bacterial Viability Kit following DEP capture at 3000 kHz from different organs (gills, stomach, and gut) of the Kuruma shrimps. Green and red fluorescence denote viable and non-viable bacteria, respectively. The yellow arrows indicate bacteria. Scale bars are 5 μm

Having identified the potential host-associated nature of bacteria newly detected via DEP in the Neocaridina shrimps, we extended our evaluation to a more complex biological system. The Kuruma shrimps, which interact with sediment, seawater, and various organisms (Macusi et al. 2022), were selected as a model due to their larger size and ecological diversity and provide an opportunity to test the capability of DEP to isolate bacteria from distinct organs. Before analyzing bacterial diversity, we evaluated whether the optimized DEP frequency of 3000 kHz maintained the viability of bacteria in samples obtained from specific organs of the Kuruma shrimps, including the gills (left and right), stomach, and gut. Consistent with the findings in the Neocaridina shrimps, the DEP-captured samples from all organs showed a significant impurity reduction and comprised viable bacteria, as indicated by green fluorescence (Fig. 2). These results demonstrate the effectiveness of DEP in isolating bacteria while maintaining viability in complex biological samples.

After confirming the ability of DEP to reduce impurities and isolate viable bacteria in Kuruma shrimp samples, we compared bacterial composition and diversity using nanopore sequencing (Table S1). The results revealed that DEP enabled the detection of bacterial genera not identified in the centrifuged samples across all evaluated organ samples (Table 2 and Table S2). Specifically, seven genera were newly detected in the left gill, one in the right gill, fourteen in the gut, and four in the stomach. Among the newly detected bacteria, *Aliiroseovarius* (Zhou et al. 2024), *Atopobacter* (Zhao et al. 2018), *Marinoscillum* (Li et al. 2023a), *Maritimibacter* (Soo and Bhassu 2022), *Roseovarius* (Niu et al. 2024), *Rhodovulum* (Koga et al. 2022), and *Shimia* (Chen et al. 2024) have been reported as beneficial bacteria in shrimp, contributing to pathogen inhibition, immune enhancement, and growth promotion.

**Table 2:**
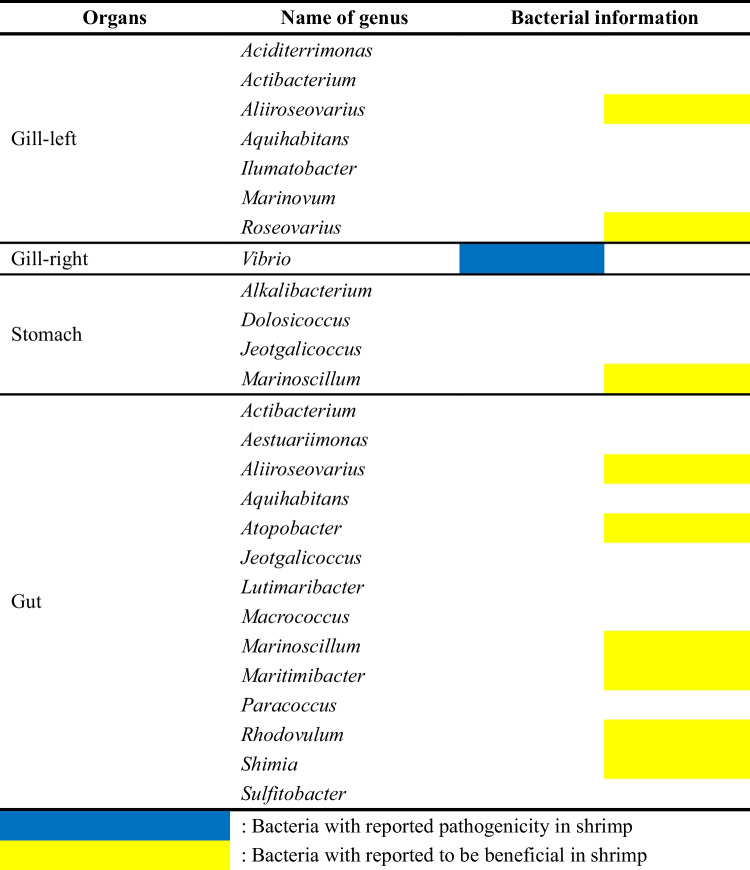
Information on newly detected bacteria after DEP capture from different organs (gills, stomach, gut) of the Kuruma shrimps

Surprisingly, *Vibrio*, often associated with shrimp pathogenicity (Sotomayor et al. 2019), was also newly detected via DEP. However, despite its common association with pathogenicity, *Vibrio* showed high dominance in the centrifuged samples of the stomach and gut, with respective abundances of 33.0% and 67.0%. Interestingly, when DEP was applied, we observed a reduction in the abundance of this bacterium, with levels dropping to undetectable in the stomach and decreasing by 21.5% in the gut (Table S2). Since DEP isolates bacteria based on their viability, this finding indicates that a portion of the *Vibrio* population may be metabolically inactive or non-viable. A previous study explored the relationship between the total *Vibrio* abundance and its metabolically active populations in shrimp guts (Garibay-Valdez et al. 2020) in which the authors suggested that while *Vibrio* might dominate the shrimp gut microbiota, the activity of this genus could be regulated by the surrounding microbiota, thereby establishing a coexistence mechanism that minimizes its impact on the host. Consistent with this, our observation of metabolically inactive *Vibrio* suggests that this genus may adopt dual roles within the shrimp digestive ecosystem. Depending on environmental conditions, *Vibrio* may act as an opportunistic pathogen or contribute to maintaining bacterial balance within the digestive microbiota. This dual functionality highlights the ecological complexity of *Vibrio* and its context-dependent interactions within shrimp.

In addition to bacteria that have shown association with shrimps, we also identified those that have not yet been linked to specific interactions with shrimp using DEP. *Sulfitobacter*, which promotes host growth in sea cucumbers (Yu et al. 2023), and *Paracoccus*, known to produce antimicrobial compounds in butterflyfish (Reverter et al. 2017), are bacteria that have been reported to support growth or play a role in immunity of the hosts. *Actibacterium* and *Ilumatobacter* (phylum Actinobacteria) and *Aquihabitans* and *Lutimaribacter* (family Rhodobacteraceae) belong to bacterial groups generally associated with producing antimicrobial substances and maintaining host physiological balance (Shijila Rani et al. 2022; Liu et al. 2019). Although specific evidence is lacking, their broader taxonomic characteristics and ecological roles suggest that these genera may contribute to pathogen suppression and growth promotion in shrimp. As for the remaining bacteria, the lack of available research made it challenging to infer their potential host interactions. Notably, *Aestuariimonas* (Park et al. 2018), *Dolosicoccus* (Collins et al. 1999), and *Marinovum* (Pradella et al. 2010) are genera with only one strain reported to date.

This study suggests that low-abundance bacteria newly detected via DEP may have the potential to play significant ecological roles in host interactions, including both pathogenicity and beneficial effects. Identifying these bacteria is crucial for understanding the host-microbiota relationships in aquatic environments and for applying these findings in practical aquaculture solutions. For instance, early detection of pathogenic bacteria could aid in developing defense systems to prevent disease outbreaks in aquaculture facilities, enhancing pathogen-focused management strategies (Wright et al. 2023). Conversely, identifying beneficial bacteria presents opportunities to improve aquaculture productivity and stability by leveraging their ecological functions (Muthu et al. 2024). These functions include pathogen suppression, enhancing host immunity, and optimizing aquaculture environments through processes like nitrogen fixation (Lo et al. 2022; Wang et al. 2020). Thus, the ability for DEP to detect low-abundance bacteria obscured by impurities provides insights for advancing pathogen management strategies and harnessing the ecological potential of beneficial bacteria in aquaculture.

### DEP Promotes the Cultivation of Potential Host-Associated Bacteria for Further Applications

**Fig. 3:**
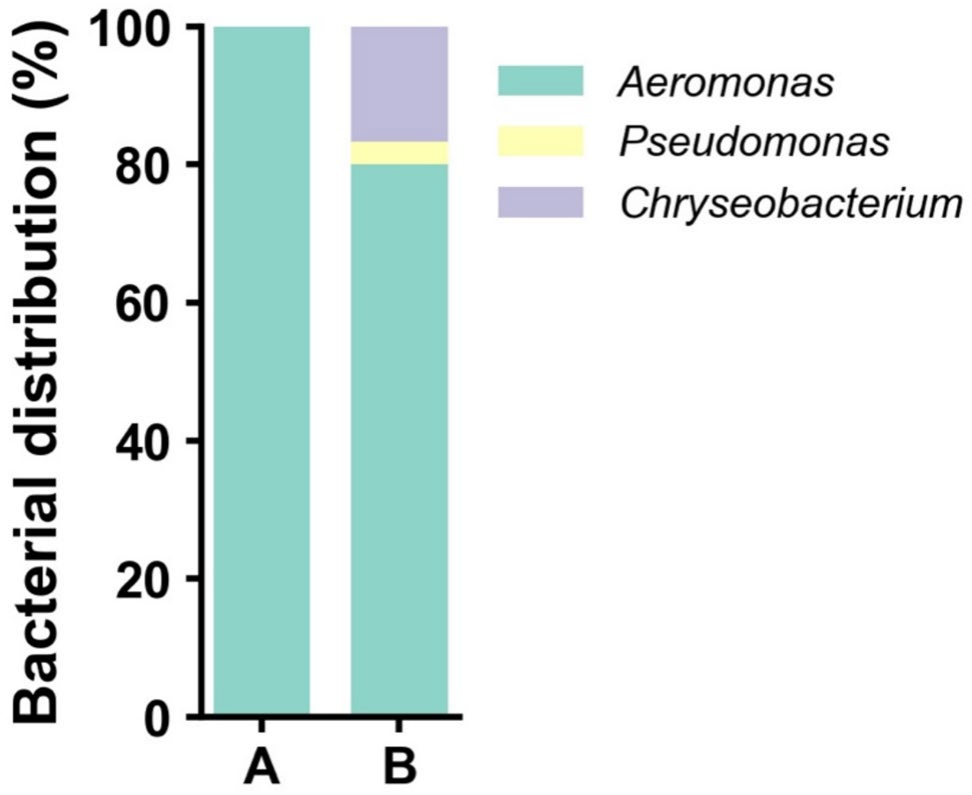
Comparison of bacterial distribution cultivated from Neocaridina shrimp samples. A, Centrifuged sample; B, DEP-captured sample

The use of DEP for impurity reduction not only enables the detection of low-abundance bacteria obscured by impurities but also highlights its potential for isolation of viable and potentially important strains applicable in culture-based research. Cultivation is fundamental for understanding bacterial physiology and host interactions, which is crucial for practical applications in aquaculture systems. To evaluate the cultivability of bacteria isolated via DEP, samples from the Neocaridina shrimps were cultured in TSB medium, commonly used for shrimp-related bacterial cultivation (Lee et al. 2024; Chumpol et al. 2017). The results revealed that only *Aeromonas* was successfully cultivated from the centrifuged sample, and it accounted for 80% of the colonies cultivated from the DEP-captured sample (Fig. 3). Interestingly, DEP enabled the cultivation of *Chryseobacterium* (16.7%) and *Pseudomonas* (3.3%), which were neither cultivated nor detected in the centrifuged sample (Table 1 and Fig. 3). These findings demonstrate that DEP not only aids in detecting new bacteria but also enhances their cultivation potential by effectively reducing impurities.

Despite being able to cultivate new bacteria upon DEP treatment, several with reports of successful cultivation in TSB medium, such as *Acidovorax* (Siani et al. 2021), *Cloacibacterium* (Nouha et al. 2016), and *Flavobacterium* (Li et al. 2021), were not cultivated in this work. We hypothesized that such a result could be due to several uncontrollable or unknown factors required by bacteria that are unique to the environment or associated with the host. Since this study primarily focused on evaluating the effectiveness of DEP in detecting low-abundance bacteria obscured by impurities, further optimization of the culture conditions for these bacteria was not pursued. Nevertheless, despite the limited scope of sample and culture conditions, DEP demonstrated its potential as a tool to promote culture-based bacterial research. Future studies should build on these findings by optimizing culture conditions for bacteria isolated via DEP and evaluating their physiological characteristics and ecological roles across diverse environments and hosts.

### Comparison with Other Approaches for Low-Abundance Bacterial Isolation

Traditionally, bacterial isolation from environmental samples has relied on culture-based approaches. These approaches have been essential for studying bacterial physiology, metabolic activity, and host interactions (Bilen 2020). However, a major limitation is that most environmental bacteria cannot be cultivated under standard laboratory conditions (Yan et al. 2023). Additionally, the isolation of low-abundance bacteria often requires extensive optimization of culture conditions, making the process time-consuming and inefficient. To overcome these limitations, various single-cell isolation techniques have been developed in recent years, including microdroplet-based technology, fluorescence-activated cell sorting (FACS), and Raman-activated cell sorting (RACS). Each of these techniques has distinct advantages and limitations depending on the specific research objectives.

Microdroplet technology enables screening and single-cell analysis by encapsulating individual bacterial cells within droplets (Hu et al. 2021). This approach allows the parallel processing of diverse bacteria in precisely controlled microenvironments. However, a significant challenge in this approach, particularly with low-abundant bacteria, is the difficulty of selectively isolating a target bacterium (Saito et al. 2021). This is because the droplet carrying the target bacterium needs to be identified among thousands or millions of others. Thus, efficient sorting and recovery of the target-containing droplets may require advanced microfluidic designs and high-resolution analytical systems. On the other hand, FACS relies on fluorescence markers to label or tag target bacteria based on their specific physiological or genetic traits (Robinson et al. 2023), while RACS identifies bacteria through unique Raman spectroscopy signatures that reflect their biochemical composition (Jing et al. 2022). Despite their advantages, both techniques require prior knowledge of target bacterial characteristics, limiting their applicability for isolating novel low-abundance bacteria from environmental samples (Robinson et al. 2023).

Compared to these techniques, DEP offers several distinct advantages. DEP separates bacteria based on their dielectric properties rather than relying on genetic or biochemical markers, allowing the isolation of previously uncharacterized bacterial species without the need for labeling. Additionally, rather than sorting individual cells at the single-cell level, DEP can isolate viable bacterial populations from environmental samples containing impurities. This ability differentiates DEP from single-cell techniques, such as microdroplet technology, as it can refine bacterial communities and facilitate the isolation of low-abundance bacteria from complex environmental samples. However, DEP also has its limitations. The efficiency of bacterial separation is influenced by the electrophysiological characteristics of the cells, which may lead to preferential isolation of bacteria with specific electrical properties. Moreover, compared to high-throughput techniques such as FACS and RACS, which can process thousands of cells per second, DEP has a relatively lower throughput, making it less suitable for large-scale screening applications.

Taken together, multiple approaches exist for isolating low-abundance bacteria, each with distinct trade-offs in terms of throughput, specificity, and prior knowledge requirements. DEP presents a unique alternative that circumvents several limitations associated with culture-based and single-cell isolation techniques, making it a promising tool for detecting and isolating low-abundance bacteria. We believe that improving DEP’s throughput and selectivity while exploring its integration with other isolation techniques could further enhance the precision and efficiency of bacterial separation.

## Conclusion

This study demonstrated the potential of DEP as a practical tool for isolating and analyzing low-abundance bacteria from complex environmental samples. By optimizing capture frequencies, DEP successfully separated bacterial fractions while reducing impurities, enabling the detection of bacterial genera overlooked by conventional methods and expanding opportunities to investigate host-microbial interactions. In addition, the maintained viability of bacteria isolated through DEP highlights their potential suitability for culture-based research and practical applications. While this study focused on validating the utility of DEP for detecting low-abundance bacteria, future research will focus on directly cultivating these bacteria to enable further investigation and application. Overall, we believe that DEP could serve as a promising approach for addressing challenges in low-abundance bacterial detection and contributing to the advancement of microbial studies across diverse ecosystems.

## Supplementary Information

Below is the link to the electronic supplementary material.

Supplementary file1 (DOCX 27 KB)

Supplementary file2 (XLSX 13 KB)

## Data Availability

The original data presented in the study were deposited in the DDBJ Sequence Read Archive under BioProject accession number PRJDB19835 and Run accession numbers DRR625164-DRR625173.
