# Supplementary material for: Dielectrophoresis for Isolating Low-Abundance Bacteria Obscured by Impurities in Environmental Samples
Source: Mar Biotechnol (NY). 2025 Mar 14;27(2):64. doi: 10.1007/s10126-025-10441-0 (PMC11909046; doi:10.1007/s10126-025-10441-0)
Supplement: Supplementary file 1 — Supplementary file1 (DOCX 27 KB) [file 10126_2025_10441_MOESM1_ESM.docx]

***Supplementary Materials***

**Dielectrophoresis for Isolating Low-Abundance Bacteria Obscured by Impurities in Environmental Samples**

**Jaeyoung Yu^1^, Hajime Yuasa^2^, Ikuo Hirono^2^, Keiichiro Koiwai^2^, Tetsushi Mori^1^***

^1^ Department of Biotechnology and Life Science, Tokyo University of Agriculture and Technology, 2-24-16 Naka-cho, Koganei-shi, Tokyo 184-8588, Japan.

^2^ Laboratory of Genome Science, Tokyo University of Marine Science and Technology, Konan 4-5-7, Minato-ku, Tokyo 108-8477, Japan

**^*^ Corresponding Author: Tetsushi Mori**

(Phone) +81-42-388-7641

(E-mail) moritets@go.tuat.ac.jp

**^†^ Co-corresponding Author: Jaeyoung Yu**

(E-mail) caguely@gmail.com

**Table S1** Summary of nanopore sequencing quality and length information

|  | **Centrifuged sample** | | | | |
| --- | --- | --- | --- | --- | --- |
|  | **Neocaridina shrimp** | **Kuruma shrimp** | | | |
|  |  | **Gill-left** | **Gill-right** | **Stomach** | **Gut** |
| **Read count** | 63,836 | 53,111 | 65,395 | 92,865 | 89,498 |
| **Mean read length (bp)** | 1,454 | 1397 | 1384 | 1460 | 1462 |
| **Mean read quality** | 14.8 | 15.6 | 15.6 | 15.5 | 15.2 |
|  | **DEP-captured sample** | | | | |
|  | **Neocaridina shrimp** | **Kuruma shrimp** | | | |
|  |  | **Gill-left** | **Gill-right** | **Stomach** | **Gut** |
| **Read count** | 63,658 | 82,345 | 75,484 | 67,236 | 65,808 |
| **Mean read length (bp)** | 1,454 | 1409 | 1394 | 1465 | 1450 |
| **Mean read quality** | 14.7 | 15.5 | 15.6 | 16.0 | 15.6 |
